# Eugenol-Based Polymeric Materials—Antibacterial Activity and Applications

**DOI:** 10.3390/antibiotics12111570

**Published:** 2023-10-27

**Authors:** Anna Kowalewska, Kamila Majewska-Smolarek

**Affiliations:** Centre of Molecular and Macromolecular Studies, Polish Academy of Sciences, Sienkiewicza 112, 90-363 Łódź, Poland; kamila.majewska-smolarek@cbmm.lodz.pl

**Keywords:** eugenol, polymeric materials, encapsulation, antimicrobial, coatings, biomedical applications, anticancer treatment, wound dressing, dentistry

## Abstract

Eugenol (4-Allyl-2-methoxy phenol) (EUG) is a plant-derived allyl chain-substituted guaiacol, widely known for its antimicrobial and anesthetic properties, as well as the ability to scavenge reactive oxygen species. It is typically used as a mixture with zinc oxide (ZOE) for the preparation of restorative tooth fillings and treatment of root canal infections. However, the high volatility of this insoluble-in-water component of natural essential oils can be an obstacle to its wider application. Moreover, molecular eugenol can be allergenic and even toxic if taken orally in high doses for long periods of time. Therefore, a growing interest in eugenol loading in polymeric materials (including the encapsulation of molecular eugenol and polymerization of EUG-derived monomers) has been noted recently. Such active macromolecular systems enhance the stability of eugenol action and potentially provide prolonged contact with pathogens without the undesired side effects of free EUG. In this review, we present an overview of methods leading to the formation of macromolecular derivatives of eugenol as well as the latest developments and further perspectives in their pharmacological and antimicrobial applications.

## 1. Introduction

**Scheme 1:**
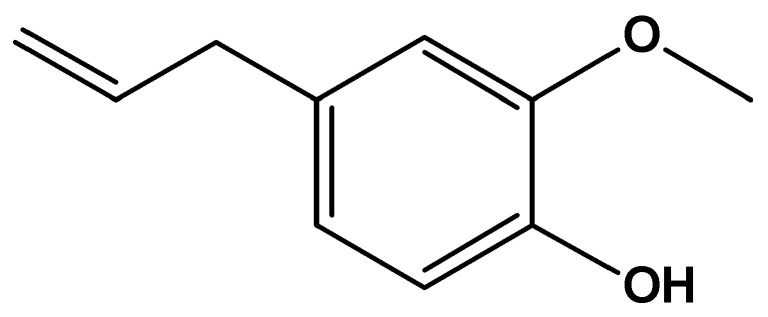
Chemical structure of 4-allyl-2-methoxyphenol.

Eugenol (4-allyl-2-methoxyphenol, EUG) (Scheme 1), a volatile naturally occurring phenol, is present in extracts of many botanical species. EUG has been identified in essential oils of cinnamon, bay and tulsi leaves, turmeric, nutmeg, and the most eugenol-rich clove *Syzygium aromaticum* (45–90% of 4-allyl-2-methoxyphenol) [1,2]. The antimicrobial, antifungal, antioxidant, anticancer, anti-inflammatory, analgesic, repellent, and insecticidal activities of this compound are well known. Because of those beneficial features, eugenol has been employed in food processing and biomedical industries and in a wide range of pharmaceutical, cosmetic, and dental care applications [1].

**Scheme 2:**
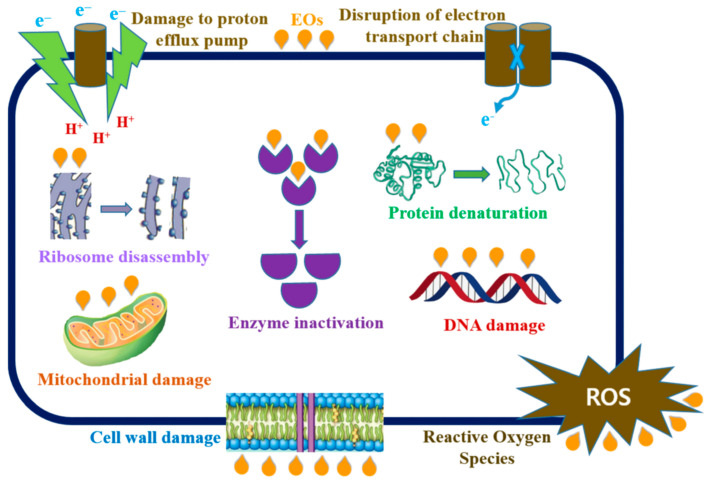
Postulated mechanism of intracellular action of essential oils on bacterial cells [4].

Bioactive compounds with antimicrobial activity, such as EUG, can be an alternative to antibiotics against many microbial pathogens, and thus reduce the increasing resistance of microorganisms to antibiotics. The minimum inhibitory concentration (MIC) and minimum bactericidal concentration (MBC) of eugenol against selected pathogenic bacteria were found to be, respectively, 0.0312–8 μg/mL and 0.0625–16 μg/mL [3]. Eugenol showed significant antimicrobial activity against *Escherichia coli* (MIC = 0.125 μg/mL; MBC = 0.250 μg/mL). The time kill curve revealed a rapid reduction in *E. coli* to undetectable levels in the presence of EUG. It was postulated that the compound alters cell membrane permeability, leading to the leakage of intracellular contents. The disruptive action of EUG on the cytoplasmic membrane was demonstrated by measurements of intracellular ATP. The MIC value of eugenol was enhanced in the presence of divalent cations, and an increase in the bacterium membrane depolarization was observed. Microscopic studies proved morphological and physiological changes in *E. coli* caused by 4-allyl-2-methoxyphenol. After membrane disruption, eugenol may further interact with the intracellular organelles causing complete damage to bacterial cells (Scheme 2).

Interestingly, the effectiveness of antimicrobials (including eugenol) depends not only on their overall concentration but also on the time used to reach that concentration. Some microorganisms may become more tolerant when treated with lower concentrations of EUG. This may be particularly important for encapsulated antimicrobial systems, where the active substances are released gradually and their minimal lethal concentration (MLC) levels are only reached after a certain period of time. Adaptation to the environmental stressor involves membrane composition changes, which make the microbial cells more resistant.

The time between antimicrobial sublethal doses was shown to have a significant effect on the efficacy population dynamics of *Staphylococcus carnosus*, *Listeria innocua*, *Escherichia coli* K12, and *Pseudomonas fluorescens* exposed to MLC of eugenol (initially, or to half doses over time) [5]. As expected, complete cell reduction occurred after immediate exposure to the full MLC dose, regardless of the type of microorganisms. The high antimicrobial efficacy of EUG on *E. coli* K12 was independent of the timing of the two doses. For other tested strains, the effect of sequential dosing varied significantly. The effectiveness of EUG against *S. carnosus* and *L. innocua* was lower the later the second half dose was applied. In the case of *P. fluorescens,* a single MLC dose of EUG reduced the number of cells below the detection level, while the sequential application of two half doses had only a bacteriostatic effect. Less severely stressed microbial populations developed resistance to EUG. To minimize such an adaptation, two consecutive half-doses should be applied fairly quickly, and exposure to low levels of stress should be avoided.

The application of several different stressors (e.g., two different antimicrobials [6] or an antimicrobial and high/low temperature [7]) simultaneously or sequentially may be a solution to avoid the phenomenon of acquired resistance. For example, the application of two antimicrobials, eugenol and lauric arginate (LAE), against *S. carnosus*, *L. innocua*, *E. coli* K12, and *P. fluorescens* was most effective if they were added simultaneously at MLC and at the beginning of the incubation period [6]. The treatment with first eugenol and then LAE at half of their MLC was efficient against *S. carnosus* and *P. fluorescens* but no sequence effects were observed against *L. innocua* and *E. coli* K12. These results were attributed to the adaptation of cells after treatment with the first active agent, which provided better protection against the second antimicrobial.

Using temperature as a second stressor, the effectiveness of treatment with a sublethal dose of EUG against *S. carnosus* LTH1502 and *E. coli* K12 C600 depended on the timing and sequence of application [7]. Differences were observed between simultaneous and sequential treatments, and also according to the type of bacteria. Initial treatment with EUG followed by a temperature change was more effective than the first temperature/later EUG option, presumably due to temperature-induced adaptation processes in cell membranes. The combined treatment was also more effective against G- *E. coli* than against G+ *S. carnosus*.

**Scheme 3:**
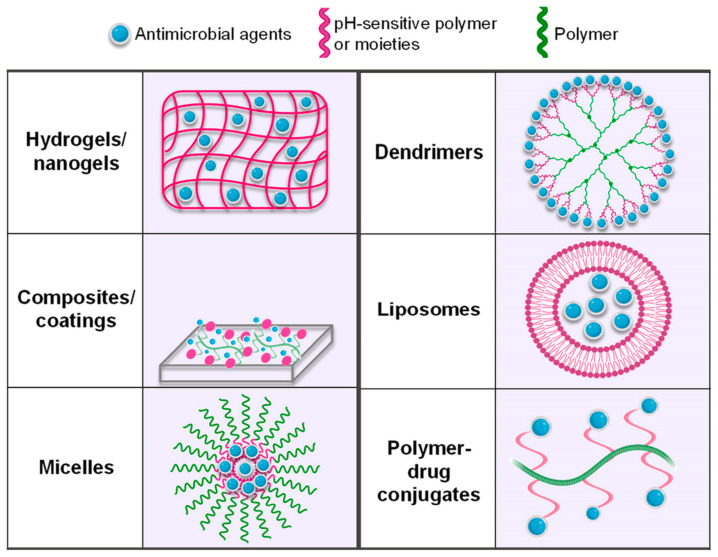
Schematic structure of macromolecular systems containing EUG (adapted from [12]).

4-Allyl-2-methoxyphenol contains three active sites in its structure: hydroxyl, allylic, and aromatic groups. It makes EUG an important starting material for the total synthesis of valuable natural products and novel drugs [1,8]. The phenol group endows eugenol not only with antimicrobial activity but also with the ability to scavenge reactive oxygen species. Apart from the bioactivity, eugenol also has significant potential as a natural, biobased building block in green technologies leading to sustainable materials. It can be obtained not only from essential oils but also from the depolymerization of lignin, and it has been used for the synthesis and modification of a wide range of polymers [9]. However, the application, processing, and storage of eugenol can be complicated due to its strong volatility, instability, and low solubility in water. The incorporation of EUG in macromolecular structures is an efficient strategy for the development and prolonged use of this volatile active substance. Polymers and composites with incorporated eugenol molecules include polyesters, polyurethanes, polyacrylates, epoxy, and polyolefins [10]. They often exhibit good barrier properties and thus have applications in the coating and packaging industries. Inexpensive, non-toxic, and environmentally friendly eugenol-based polymeric resins exhibit attractive properties such as thermal stability, water resistance, and flame retardancy [11]. Moreover, polymeric materials of different types (Scheme 3) can be used as carriers of EUG, either in their free form or bound to macromolecular structures. This article presents an overview of developments in the synthesis, pharmacological and antimicrobial properties, and applications and further perspectives of macromolecular systems containing or derived from eugenol.

## 2. Discussion

### 2.1. Eugenol-Based Polymers and Composites for High-Performance Antimicrobial Coatings

Polymers obtained with the use of eugenol are receiving growing interest because of their sustainability and enhanced physicochemical characteristics. The development of such polymeric materials designed to control bacterial biofilm growth is an important practical approach. They can be used to generate bacteria-repellent or antibacterial coatings aimed at preventing bacterial colonization. EUG-derived polymers can be used as coatings in a wide range of applications [13]. Excellent thermosetting materials were also obtained with monomers that are derivatives of eugenol [14,15]. For example, eugenol molecules were transformed into 4,4′-(butane-1,4-diyl)bis(2-methoxyphenol) by the Ru-catalyzed olefin metathesis coupling reaction [16]. The product was then converted into a polycyanurate thermoset resin that exhibited a glass transition temperature (T_g_) of 186 °C and was thermally stable above 350 °C. The water uptake of the cured material was only 1.8% after immersion in hot water (85 °C) for four days. The high hydrolytic stability makes this polymer well-suited even for maritime environments. EUG molecules can also be transformed into other monomers suitable for the free radical polymerization reaction, including EUMA—eugenyl methacrylate, and thus particles of poly(DMA-co-EUMA) (DMA—dopamine methacrylamide) were obtained via precipitation copolymerization [17]. The antibacterial activity test against *E. coli* showed a >90% antibacterial rate of PD5E5 and PD3E7 particles (5:5 and 3:7 molar ratios of DMA to EUMA). Another derivative of eugenol—4-allylpyrocatechol—was used for the preparation of photocurable polymer networks of high macroscopic adhesion to glass, marble, aluminum, and steel [18]. Eugenol was also used for the synthesis of environmentally sustainable polybenzoxazines [19], polybenzoxazine-POSS nanocomposites [20], and analogues of urushiol [21] of superior adhesion, mechanical strength, and antioxidant properties. These polymerization processes are green and do not involve the evolution of volatiles.

**Figure 1:**
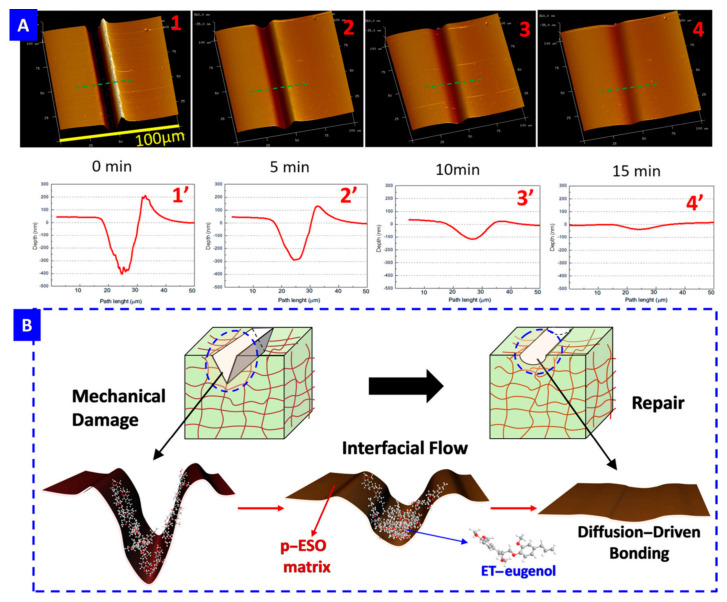
(**A**) Atomic force microscopy images of the damaged surface of ET-eugenol/p-ESO sample (mass ratio of 1:2) and the process of its thermally induced self-healing with time and (**B**) proposed explanation of the healing mechanism via the diffusion-driven bonding of ET-eugenol near the damaged interfacial region. Reprinted with permission from [26]. Copyright {2020} American Chemical Society.

Eugenol-derived polymers are also capable of self-healing, which is a very important feature regarding the stability of coating materials that affect their antimicrobial/antibiofilm performance. The process can be based either on thiol-oxidation reactions [22,23,24] or hydrogen bonding [25]. Interestingly, eugenol derivatives can take part in a self-healing process through mechanisms based on the formation of multiphase structures in polymer composites. A biobased molecular glass (ET-eugenol) obtained by thermochemical conversion of epoxidated eugenol and then incorporated into the matrix of polymerized soybean oil (p-ESO) can be an example [26]. The scratch damage on the surface of ET-eugenol/p-ESO (1:2 wt./wt.) composite was healed at 90 °C within 15 min or by UV radiation within 20″ effectively (efficiency up to 88%) and without a dimension change (Figure 1). Furthermore, the incorporation of ET-eugenol improved the mechanical properties of the p-ESO matrix as comparative tensile tests of these materials showed a 2.7-fold increase in ultimate stress.

The synthesis reactions of some EUG-based polymers involved phenolic groups, which may inevitably lead to a partial loss of the antioxidant activity of eugenol. Nevertheless, several systems of this kind showed significant antimicrobial activity. For example, water-insoluble polyeugenol of a high molecular mass (~10 kg mol^−1^, DP = 60) was synthesized by cationic polymerization and showed antibacterial activity with a slow response against *E. coli* and *S. aureus* [27]. Polyeugenol of a high molecular weight (~800–2200 kg mol^−1^) was also tested against *S. aureus* and *E. coli* [28]. The antibacterial action was evaluated with the well diffusion method at 1, 2, 3, 4, and 5% concentrations to observe inhibition zones of 17.42, 17.76, 18.79, 21.42, and 22.55 mm for *S. aureus* and 15.87, 17.23, 17.56, 18.24, and 19.21 mm for *E. coli*. These results indicate that antibacterial activity was rather strong. It was supported by the strong antioxidant activity of the polymer indicated in the tests against free radical DPPH (2,2-diphenyl-1-pycrylhydrazyl), which resulted in an IC_50_ value of 80.47 µg/mL.

**Figure 2:**
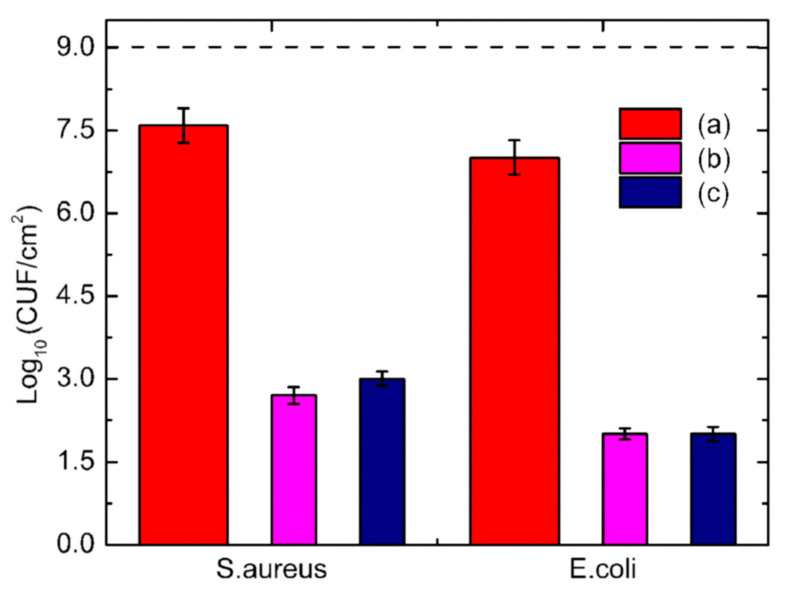
Concentration of *S. aureus* and *E. coli* on (a) as-received stainless-steel supports, (b) immersed for 45 min in liquid eugenol, and (c) coated with EDF for 45 min (—bacteria concentration in the initial inoculum) [29]. Copyright (2021), with permission from Elsevier.

Eugenol-derived polymeric films (EDF) can be deposited on stainless-steel surfaces using atmospheric pressure plasma discharge [26]. Scanning electron microscopy showed that the entire surface of the substrate was covered with circular structures of ~10–20 µm in diameter. FT-IR spectra showed that the hydroxyl and aromatic groups, which are key features for the antibacterial activity of native eugenol, were preserved. An increase in the hydrophilicity of the surface after the deposition was revealed. EDF deposits can be potentially of use in coating processes for biocompatible materials. The adhesion and proliferation of *E. coli* and *S. aureus* on EDF surfaces were inhibited by more than 78 and 65%, respectively, in comparison with control polystyrene plates (Figure 2).

**Figure 3:**
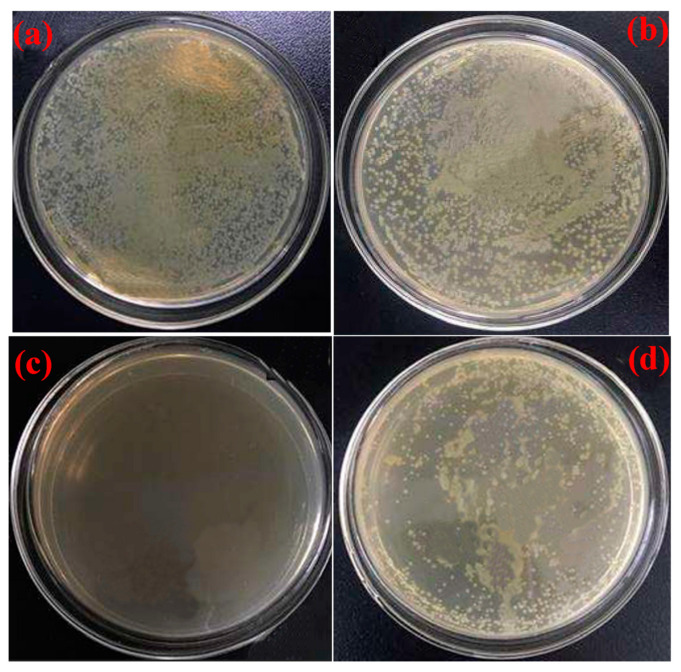
Antimicrobial activity presented by changes in agar plate cultures: The blank group (**a**), HMQ (**b**), eugenol (**c**), and BPMQ (**d**) [30].

Eugenol was also used for the chemical modification of inorganic and organic polymeric materials. Hydrosilylation of the alkyl group in eugenol with hydrogen-containing MQ silicone resin (M: monofunctional and Q: tetrafunctional siloxane units) resulted in a hybrid bio-phenol MQ silicone resin (BPMQ) [30]. The thermal stability of BPMQ was significantly improved. The maximum degradation rate increased from 250 °C (MQ precursor) to 422.5 °C (BPMQ) due to the modification with EUG. The mass of residual yields left at 600 °C increased accordingly, from 2.0% (MQ) to 28.3% (BMQ). The resin was also investigated for its antibacterial properties against *E. coli*, markedly enhanced as a result of EUG grafting (Figure 3). Hybrid materials of this type can be used for biomedical silicone rubber products or pressure-sensitive adhesives.

As an example of organic macromolecules grafted with EUG, the eugenol-methacrylate monomer with the free phenol group preserved was polymerized and copolymerized at different concentrations with 2-hydroxyethyl methacrylate (HEMA) [31]. The obtained amorphous homopolymer (pEUMA) and copolymers (pHEMA-EUMA) were equally thermally stable as pHEMA but much less swellable in water. Flexible pEUMA exhibited good elastic properties, even at high deformation frequencies (up to 80 Hz). Owing to the presence of phenol functions, the polymers acted as radical scavengers and showed antimicrobial activity towards *Staphylococcus epidermidis*. Bacterial growth was gradually reduced by increasing the EUMA content to 30% (~20% bacterial growth (BG) vs. 100% BG for the control sample and pHEMA). Nevertheless, higher amounts of the eugenol derivative did not bring further enhancement of the antimicrobial properties (~25% BG for pHEMA-EU50).

EUMA was also used for the preparation of a polymer for self-polishing antifouling coatings [32]. The self-cleaning process involved hydrolysis of the phenolic ester group with the release of eugenol molecules. As a result, the surface energy of the coating increased as the hydrophobic surface was transformed into hydrophilic. EUG was released from the coating most rapidly at the beginning of immersion in artificial seawater, but after 15 days, the process had stabilized (0.48 μg·cm^−2^·day^−1^ and 0.14 μg·cm^−2^·day^−1^, respectively, observed for the sample with the highest release rate). The resin has good antibacterial and anti-algae properties. Coatings with high eugenol content were also avoided by mussels *Mytilus edulis*.

Essential oils (EO), such as eugenol, can be also physically blended with various polymers. The structural stability of such materials and their antimicrobial performance are limited by the phase separation phenomena, especially if there are no specific interactions between EUG and the polymer matrix. For example, poly(ethylene-co-vinyl acetate) copolymer (EVA) films containing citronellol, eugenol, and linalool were prepared and evaluated for their antimicrobial and antibiofilm action against *Listeria monocytogenes*, *S. aureus*, *Staphylococcus epidermidis*, *E. coli*, and *P. aeruginosa* (mono- and dual-species tests) [33]. The addition of the essential oils influenced the mechanical properties of EVA (15% elastic modulus decrease, 30% tensile strength decrease, 10% elongation at break increase). EVA/citronellol and EVA/eugenol at 7 wt% of the essential oils content showed the best inhibition of bacteria growth (30–60% and 15–30%, respectively, after 24–48 h of incubation). However, in both cases, the inhibition decreased after 240 h of incubation. Antibiofilm action of the composites was strong, even after prolonged incubation time. A 40–70% decrease in the bacterial biomass of *L. monocytogenes*, *S. aureus*, and *E. coli* was found for the EVA/EO composites in comparison with the pure EVA control. Interestingly, better results were achieved with EVA/eugenol than EVA/citronellol when inoculated simultaneously with combinations of *S. aureus* and *E. coli*. The biomass accumulated was higher (EVA + citronellol) or lower (EVA + eugenol) than that in monoculture biofilms. Similar findings were obtained with measurements of the metabolic activity of viable cells with 2,3-bis [2-methyloxy-4-nitro-5-sulfophenyl]-2H-tetrazolium-5-carboxanilide assay.

Eugenol can be also used to modify natural biopolymers or green synthetic polymers obtained from renewable resources, such as chitosan, with well-known antimicrobial and therapeutic activity. A chitosan-based polybenzoxazine monomer was synthesized in water and then crosslinked by thermal treatment via ring-opening polymerization to form free-standing polymer films [poly(E-Ch)] [34]. Hydrogen bonding interactions between chitosan and polybenzoxazine macromolecules resulted in an enhancement of the thermal and mechanical properties (T_10_ of ~260 °C; ε′ of ~3.6 GPa; and T_g_ of ~135 °C). The poly(E-Ch) was found to be effective in preventing bio-film-associated infections (*S. aureus* and *Candida albicans*). Films made of chitosan and poly(vinyl alcohol) (PVA) (ratio 30/70 *w*/*w*; 9 wt%) were prepared by the solvent casting and phase inversion method and then supplemented with 1 and 10 wt% of EO [35]. The film thickness and degree of swelling increased with the EO content. The chitosan films alone exhibited antimicrobial properties and were able to eradicate *P. aeruginosa* within one hour. Yet, chitosan/PVA films loaded with EUG showed significantly improved antimicrobial action within 2 h of contact, particularly against *S. aureus*, compared to the corresponding unloaded samples. Such materials could potentially be used in the treatment of chronic wounds.

Eugenol was also grafted to the copolymer of carboxymethyl cellulose (CMC) and cysteamine hydrochloride (CYST) by the thiol-ene click reaction between the allyl group of EUG and mercapto-groups of CYST [36]. The resulting amphiphilic polymer (CMC-CYST-EUG) showed a 7.8-times longer duration of antimicrobial activity compared to pure EUG under the same environmental conditions. Cytotoxicity tests show that the polymer is safe at low concentrations. CMC-CYST-EUG can also increase the shelf life of fruits and vegetables owing to their good adhesion. The efficacy of CMC-CYST-EUG in pest control was significantly higher than that of EUG.

In summary, inorganic and organic macromolecular systems functionalized with eugenol, both as a building block and as a doping additive, show interesting physicochemical, mechanical, and antimicrobial properties. This approach increases the potential contact time of eugenol with pathogenic micro-organisms and increases the efficiency of their inactivation. Of particular interest is the possibility of using eugenol for antimicrobial modification of ‘green’ biopolymers.

### 2.2. Eugenol-Based Materials for Food Packaging

Bioactive and biodegradable hybrid materials are a very interesting solution for, e.g., environmentally friendly food storage [4,37,38]. Packaging technologies have to meet growing demands regarding the extended shelf-life and good quality of products, especially fresh or low-processing food. The improvement of food preservation via the design of new technologies incites growing interest in biocompatible and preferably biodegradable antimicrobial/antibiofilm materials. The process of making “green” polymeric materials for active packaging is related both to the type of matrix and the incorporated additives.

It was shown in the tests performed with selected bacterial strains isolated from dairy products (*E. coli*, *S. aureus*; *Bacillus pumilus*, *Bacillus subtilis*, *Bacillus tequilensis*, and *Stenotrophomonas maltophilia*) that biodegradable polymer films of poly(lactic acid) (PLA), poly(butylene adipate-co-terephthalate) (PBAT), and poly(butylene succinate) (PBS) have a varying propensity for biofilm growth [39]. Neat PLA was less prone to biofilm settlement than PBS. The antimicrobial activity and the reduction in biofilm formation on polymer films were improved upon the inclusion of thymol and eugenol into these materials. Biofilm formation was not detected on polymer films with incorporated antimicrobial compounds.

On the other hand, polylactide-based materials covalently grafted with eugenol did not exhibit high antimicrobial action [40]. The polymers were synthesized by homopolymerization reactions of eugenol-bearing L-malic acid-derived O-carboxyanhydride (L-MalOCA) or its copolymerization at two different ratios with the L-lactic acid-derived OCA (L-LacOCA) monomer. The thermal properties of the resulting polymers depended on the content of L-MalOCA. The antimicrobial activity of the hybrid materials against common food-related bacteria, namely *E. coli*, *Pseudomonas aeruginosa*, *S. aureus*, and *B. cereus*, was tested. The action of the eugenol-rich poly(L-MalOCA) product was detected against *S. aureus* and *B. cereus*, and to a minor extent, against *P. aeruginosa*. No inhibition was found in tests against *E. coli*.

Eugenol, cinnamaldehyde (CIN), and carvacrol (CAR) were added to enhance the antimicrobial properties of zein-based materials [41]. Polyethylene glycol (PEG) and oleic acid (OA) were used as plasticizers to improve the mechanical properties of zein. Compared to OA, PEG efficiently improved the tensile strength and elongation (%E) of zein films but induced weaker water barrier properties. The EO-embedded zein films demonstrate a gradual release of EO (up to 96 h) and showed even better antimicrobial effects against *S. aureus* and *E. coli* than neat EO in a filter paper sample (FPS). This can be explained by the difference in release rates for EO in zein-PEG and FPS. The chemical associations lead to a controlled release of EO. EO interacts with zein through weak chemical bonds and their molecules are chemically entrapped in the zein film, thus the evaporation rate is low. The strongest antimicrobial effect was noted for CIN (16.67 mm inhibition zone against *S. aureus* for zein/PEG with 5% CIN and 4.67 mm for pure CIN). CAR was active only at a concentration of 3% in zein-PEG, and it was comparably effective toward both bacteria. Unfortunately, EUG showed no inhibitory effect against *S. aureus* and *E. coli* at the 1% and 3% concentration levels, respectively. Tests at 8% of EUG indicated that the G- bacterium *E. coli* was more sensitive to EUG than G+ *S. aureus*, either entrapped in zein-PEG or in pure form in FPS.

**Figure 4:**
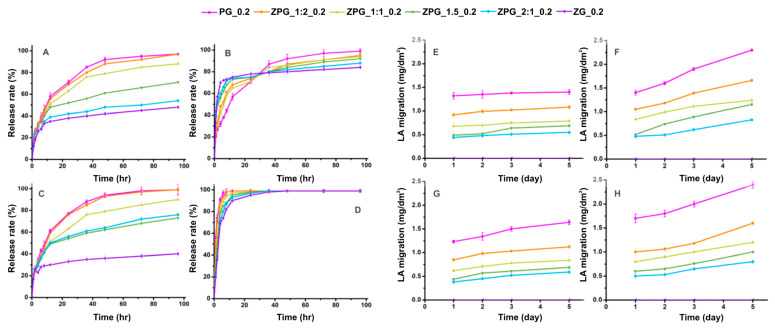
EUG migration from the PLA/zein films to (**A**) deionized (DI) water, (**B**) 3.0% (*w*/*v*) acetic acid solution, (**C**) 10% (*v*/*v*) ethanol, and (**D**) 50% (*v*/*v*) ethanol; compared to lactic acid (LA) migration under the corresponding conditions: (**E**) DI water, (**F**) 3.0% (*w*/*v*) acetic acid solution, (**G**) 10% (*v*/*v*) ethanol, and (**H**) 50% (*v*/*v*) ethanol. Ref. [42] Copyright (2022), with permission from Elsevier.

Eugenol was also added to improve the antimicrobial properties of a blend of PLA and zein of improved mechanical and barrier properties (elongation, tensile strength, water vapor, and gas permeabilities) [42]. EUG significantly inhibited the growth of both *S. aureus* and *E. coli*. The migration tests of eugenol from the polymer composites into distilled water and three food simulants (10% (*v*/*v*) ethanol simulating hydrophilic food, 50% (*v*/*v*) ethanol for lipophilic products, and 3.0% (*w*/*v*) acetic acid solution for acidic hydrophilic products with pH < 4.5) were conducted (Figure 4). Interestingly, the results illustrated the different actions of various types of food on the migration rates of eugenol. The EUG migration rate was controlled by zein. In the case of hydrophilic food, it was generally faster for materials with a higher content of PLA, and transport of EUG in this case was slowed down by zein nanoparticles. EUG molecules moved most rapidly in the environment simulating lipophilic products. In such systems, hydrophobic interactions of both zein and eugenol with the stimulant exist. The migration of lactic acid (LA), that is the degradation product of PLA, to water and three different food simulants depended on the content of PLA, but the amount was generally low (<2.5 mg/dm^2^ in 5 days). This demonstrates the lack of degradation of the polylactide by eugenol.

Gelatine (GL)/chitosan (Ch)-based active, biodegradable, and edible films and coatings containing eugenol and oregano (*Origanum vulgare*) essential oil (OEO) in different OEO:EUG ratios were designed for food preservation [43]. Retardation of the oxidative processes and the growth of pathogenic microorganisms (*S. aureus* and *E. coli*) on the surface of food (e.g., fresh cheese) could be achieved. The optimal activity of systems containing 0.50% EUG  +  0.50% OEO and 0.25% EUG  +  0.75% OEO is attributable to the synergistic antimicrobial effect of EUG, OEO, and Ch. The water vapor barrier properties in OEO-containing films were markedly enhanced despite a slight phase separation in the hybrid materials, especially with the high OEO:EUG ratio. The observed higher moisture content and water solubility values can be related to the morphology of those coatings. The optimal film structure (a sample with 0.5% wt/wt of OEO  +  0.5% wt/wt of EUG content) correlated with preservation properties of coatings that were tested on fresh cheese during 14 days of storage at 4 °C. However, none of the tested coatings prevented WL (freshness) in the cheese samples.

The eugenol content of physical blends with biocompatible polymers is not high since larger amounts of EUG cannot be retained by the polymer matrix. It is thus desirable to introduce an ingredient that traps the eugenol molecules. For example, eugenol can form inclusion complexes with β-cyclodextrins (βCD). The formation of host-guest interactions was exploited for the preparation of a water-absorbent antibacterial mat, made of electrospun fibers composed of polylactic acid-β-cyclodextrin/eugenol (PLA-β-CD/EUG), a super absorbent polymer (SAP), and filter paper [44]. The mats were shown to restrain the growth of *S. aureus*, *E. coli*, *P. aeruginosa*, *L. monocytogenes*, *Bacillus pyocyaneus*, and *Salmonella typhimurium*. Comparative EUG release tests from PLA-β-CD/EUG and PLA-EUG fiber membranes demonstrated that the maximum release time for the latter was 4-fold lower. Stable release profiles were then observed for both samples over a period of up to 40 min. The slower and steady EUG release profile of PLA-β-CD/EUG fiber membranes can be attributed to host–guest interactions between EUG and β-CD. In addition to the demonstrated inhibitory effect on both G+ and G- bacteria, the water-absorbent antibacterial PLA-β-CD/EUG mats had a longer-lasting EUG release effect in different food simulants. The slowest eugenol delivery was found for oily food, which may be promising in meat preservation.

Fish gelatine mats containing cinnamaldehyde (CEO), limonene (LEO), and eugenol at 1, 3, and 5% with β-cyclodextrins at an equimolar ratio were prepared from electrospun fibers [45]. It was expected that the diverse structure of EO molecules (aldehyde, terpene, and phenol derivatives) would cause different interactions with βCD. The presence of essential oils did not disturb the morphology of fibers (defect-free) but reduced their mechanical properties (tensile strength and elongation at break), despite the improvement in the gelatine melting temperature by 3 to 13 °C. Although all three types of gelatine mats showed similar antifungal activity at 5% concentration of essential oils, their antibacterial properties depended on the type of EO additive. The highest radical scavenging activity (96.5%) was obtained with EUG, while cinamaldehyde resulted in the highest inhibition zone (27.73 mm). The functional stability of the developed mats and a slow release of EOs for 60 days at ambient temperature were noted, which indicates the applicative potential as active antimicrobial packaging for low-to-intermediate moisture foods.

Packaging materials with EUG molecules built into the macromolecular system were also obtained. Polymacrolactones with pendant eugenol moieties were prepared via the functionalization of poly(6HDL-*co*-PDL) copolymers of random structure that were obtained in ring-opening copolymerization of ω-6-hexadecenlactone (6HDL) and ω-pentadecalactone (PDL) carried out in the presence of a pyridylamidozinc(II) complex [46]. Eugenol was converted into 4-(3-mercaptopropyl)-2-methoxyphenol (TE) and then grafted by thiol-ene addition to the unsaturated C=C bonds of 6HDL units in poly(6HDL-co-PDL). The structure and thermal properties of the final products were modulated by the amount of eugenol-derived side groups. The antimicrobial activity of TE-functionalized copolymers against *E. coli* was also dependent on the content of TE units in the films.

Copolymers of eugenol-trithiol methylenebisacrylamide (E3TMBA) and eugenol-trithiol, allyl eugenol (E3TAE) of high thermal stability (5.0% mass loss at 179 °C for E3TMBA and 255.9 °C for E3TAE) were synthesized via solvent-free photopolymerization of eugenol and trithiol with methylenebisacrylamide (MBA) and allyl eugenol (AE) as crosslinkers [47]. E3TMBA and E3TAE were moderately hydrophilic (contact angle values (θ) of 74° and 58.4°, respectively). They exhibited glass transitions at 48 °C and 30 °C, respectively. The trend correlated with their mechanical properties (tensile strength of 0.463 MPa and a 6% elongation recorded for E3TMBA and 0.378 MPa/33% for E3TAE). The antibacterial activity against *E. coli* (12/24 h inhibition values: 46.65%/77.04% (E3TMBA) and 55.7%/83.04% (E3TAE)) and *S. aureus* (12/24 h inhibition values: 64.11%/82.06% (E3TMBA) and 68.8%/80.04% (E3TAE)) was good, which is promising for their food packaging potential.

Eugenol and three dithiols of various structures were used for the preparation of macromolecular antioxidants (MAOs), subsequently applied as additives to poly(vinyl alcohol) (PVA) [48]. The polymer main-chain morphology and properties were tuned by the structure of dithiols. Mono-phenolic and catechol moieties from EUG were distributed along the polymer backbone, with coexisting thioethers, oxyethers, or vicinal diol groups. Macromolecules with catechol groups and vicinal diols exhibited a synergistic effect to enhance the anti-oxidation storage stability. Solution-cast hybrid films exhibited enhanced mechanical strength and toughness. Excellent scavenging of DPPH radicals was noted for PVA containing ≥5% MAO (at 7.5% MAO complete scavenging after 3 min).

For packaging applications, eugenol can be also used in the neat form, for physical modification of polymeric materials. Their final properties depend on the processing conditions. For example, EUG was added to linear low-density polyethylene (LLDPE) films under conditions of supercritical CO_2_-assisted impregnation [37]. The effect of this process can be modulated by the pressure and depressurization rate. Binary mixtures of EO containing thymol, carvacrol, citral, and eugenol were also introduced into polypropylene (PP)/polyamide (PA)/nanoclays composite blends processed into thin films [49]. Their release kinetics were studied in correlation with their capacity to inhibit the growth of *E. coli* and fungi over time. The inhibition of fungal growth on food samples for over 50 days indicated the potential use of the developed EO-loaded films as active food packaging.

The above examples prove that the application of eugenol for chemical and physical modification of various polymeric materials of synthetic and natural origin can yield prolonged beneficial antimicrobial activity. These hybrid compositions can be used for packaging applications (thin films and adsorbent mats). Despite previous observations of bacterial adaptation to the low concentration of eugenol, such effects were not retained on an experimental time scale in polymeric materials that release EUG slowly.

### 2.3. Encapsulation of Eugenol in Polymeric Materials for Biomedical Applications

Increasing antibiotic resistance of bacterial strains necessitates the development of new antibacterial agents and therapies to prevent uncontrolled disease-causing pathogenic microorganisms. The increasing resistance of bacterial strains to antibiotics requires the development of new antimicrobial agents and therapies to prevent pathogenic microorganisms from causing disease uncontrollably.

Nano-based and combinatorial strategies offer solutions for reducing the use of antibiotics. The toxicity of various inorganic and organic antibacterial compounds has caused increasing interest in medicinal plant extracts with biocompatible and potent antibacterial characteristics. Such green antimicrobials are volatile and most often insoluble in water; thus, their bioavailability is rather poor. Nano-scale technologies provide a range of solutions to the problem of their poor bioavailability as well as oxygen sensitivity. As an additional result, the therapeutic potential of the active compounds may be enhanced [50,51,52,53,54]. Bioactive phytochemicals can be encapsulated or entrapped within inorganic or organic hydrophilic capping agents to improve their solubility and stability. Various nanocarriers, including nanoemulsions, dendrimers, micelles, liposomes, solid lipid nanoparticles, and nanoparticles of biodegradable polymers, can be employed for delivery of the active agents. For example, zein, sodium caseinate (NaCas), and pectin were exploited as polymeric matrices to formulate colloidal complex nanoparticles for the encapsulation of eugenol [55]. Small (~140 nm under optimized conditions), uniform, and stable particles were obtained. The process was affected by synthetic conditions and, preferably, pH 6.6 corresponding to the isoelectric point of zein should be maintained.

Analogously, pH-responsive biopolymeric nanocapsules containing essential oils of *Thymus vulgaris*, *Rosmarinus officinalis*, and *Syzygium aromaticum* were formed with the use of chitosan, alginate-chitosan, guar gum-chitosan, xanthan gum-chitosan, and pectin-chitosan [56]. The average encapsulation efficiency in nanovessels of 100–500 nm was 60% while the loading efficiency was 70%. Essential oil release kinetics were determined in pH = 3, 5.6, and 7.4. The best results were obtained for nanocapsules built from a chitosan-guar gum structure. The chitosan-guar gum and chitosan-pectin nanocapsules released 30% of essential oils (similar release kinetics for thymol, eugenol, and α-pinene) at pH 3 and 80% at pH = 7.4 during 3 h. Growth rates of *S. aureus* and *E. coli*, as well as their minimal inhibition concentration values, were estimated. The MIC of *Thymus vulgaris* and *Syzygium aromaticum* essential oils ranged from 0.025 to 0.5%.

Release kinetics of thymol, cinnamaldehyde, and eugenol encapsulated in liquid lipid nanoparticles (LLN) or solid lipid nanoparticles (SLN) were studied as a function of the lipid structure and carrier particle concentration in pullulan-based films [57]. The EO significantly reduced the crystallization temperature of SLN, and their release rate from SLN films was twice as high as that of LLN films. The release rate also depended on the type and amount of active compound (thymol > eugenol > cinnamaldehyde).

Eugenol and linalool were also encapsulated in polylactic acid [58]. Their antimicrobial activity against *E. coli*, *Salmonella enterica*, *S. aureus*, and *Listeria monocytogenes* was studied, and their minimum inhibitory concentration and minimum bactericidal concentration were evaluated. MBC values of these strains were, respectively, 0.39%, 3.13%, 0.78%, and 1.56% (for eugenol) and 0.39%, 12.50%, 0.39%, and 12.50% (for linalool). Inhibition zones of relevant size were detected for EUG against *E. coli* (60 mm) and linalool against *Salmonella* (32 mm). The capsules have prolonged efficiency, continuing to release the active species for up to 40 days. Such bio-based antimicrobial systems can be potentially applied as active food packaging.

**Figure 5:**
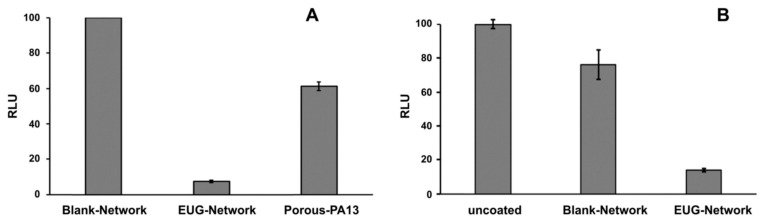
Normalized relative luminescence intensity of (**A**) media containing surface-bound MRSA and *K. pneumoniae* from monoliths of the control blank-network, EUG-containing network, and porous poly(methyl methacrylate-co-dimethylacrylamide) (PA13); and (**B**) BacTiter-Glo™ assay of surface-bound MRSA and *K. pneumoniae* on the surface of uncoated endotracheal tube, control and EUG-network. Reproduced from Ref. [59] with permission from the Royal Society of Chemistry.

The slow release of natural antimicrobials from nano-vessels can also improve the performance of bacteria-repellent polymer coatings. A hemocompatible interpenetrating network designed to repel bacteria was prepared with the use of poly(lauryl acrylate) nanocapsules containing eugenol [59]. The vesicles were entrapped within the network and EUG was slowly released. A significant reduction in surface-bound *Klebsiella pneumoniae* and methicillin-resistant *S. aureus* (MRSA) was achieved with this system (Figure 5) when the hybrid material was coated on a catheter and an endotracheal tube.

Eugenol can be also incorporated into cross-linked polymer nanocomposite “sponges” [60]. In this form, it was applied for the treatment of bacterial biofilms of *E. coli* (CD2), *P. aeruginosa* (CD1006), and the *E. cloacae* complex (CD1412). This method was found to be very effective at delivering phytochemicals containing phenyl hydroxyl groups, such as eugenol and carvacrol. The results indicated that encapsulating EUG and other phytochemicals (methyl eugenol, carvacol, linalool, (+)-limonene, p-cymene, and α-pinene) effectively eliminated biofilms while demonstrating low cytotoxicity against mammalian fibroblast cells. It makes the NC sponges a promising direction to address wound biofilm infections. MICs (*v*/*v*%) of neat eugenol against these bacterial strains were 16, whereas, in the nanocomposites, it was reduced to 4. Similar results were obtained for carvacol, whereas the action of other phytochemicals was much more reduced when incorporated into NC “sponges”. NCs loaded with eugenol (log P: 2.49) were able to kill 90% of bacteria in the biofilms at approximately 12 *v*/*v*%. Similar results were obtained in the case of EUG for MRSA (CD489) (MIC of 16 *v*/*v*% for neat eugenol was diminished to 4 for EUG-NCs), whereas no reduction in antimicrobial activity was noted for (+)-limonene, p-cymene, and α-pinene after their incorporation into NCs.

Silica nanoparticles capped with triblock copolymer Pluronic F-127 were also used as nanocarriers of eugenol [61]. Results obtained with dynamic light scattering (DLS) and zeta-potential measurements suggested that in such particles, eugenol molecules are dispersed between the polymer chains of Pluronic F-127, most likely in the region of poly(propylene oxide) blocks. Nanocarriers obtained in Pluronic F-127 emulsions were only stable in aqueous medium under conditions in which the ratio between the weight fractions of Pluronic F-127 and eugenol > 1.5. For other stoichiometries, phase separation appeared within 48 h after their preparation. The weight fraction of silica nanoparticles must be low to obtain a stable emulsion. The potential application of these nanocarriers in the development of new formulations for pest control was studied. The formulation was tested as an insecticide (up to 60% mortality of *Pediculus humanus capitis* after application of the water emulsion of EUG/silica/F-127).

#### 2.3.1. EUG-Based Materials for Anticancer Treatment

**Scheme 4:**
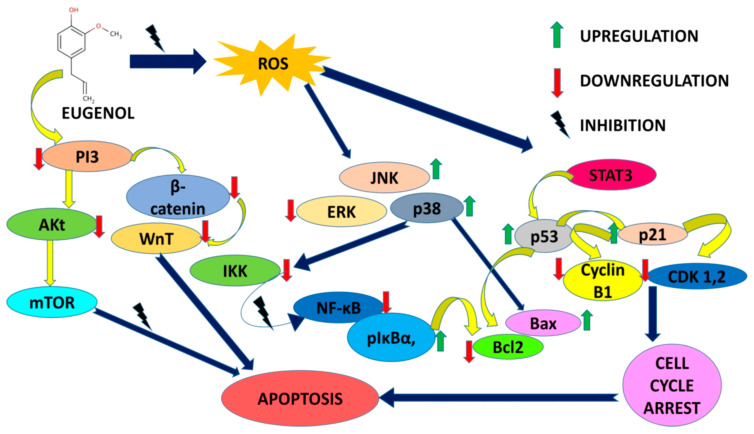
Eugenol regulated molecular signalling cascade in combating proliferation and apoptosis induction [62].

Preclinical studies indicate that, aside from the antimicrobial aspects, 4-allyl-2-methoxyphenol also has antioxidant, antimutagenic, antigenotoxic, and anti-inflammatory properties and can be effective against various types of cancer of different origins (e.g., breast, cervical, lung, prostate, melanoma, leukemia, osteosarcoma, and glioma) [62]. The pharmacological benefits include the action of eugenol at various steps of the transition from normal cells to transformed tumor cells. Eugenol acts on several biomolecular pathways including cell division cycle arrest, autophagy, and apoptosis (Scheme 4) [63,64]. It can block the expression of proinflammatory transcription and growth factors, oncogenes, anti-apoptotic and angiogenic proteins, and adhesion molecules.

The delivery of the active agent can be affected by eugenol nano-formulations that have the potential for both the treatment and prevention of cancer. Several reports presented advancements in the novel nano-drug delivery approach that can enhance the therapeutic profile of EUG and improve its antioxidant activity.

Clove oil (containing eugenol as a major bioactive constituent) nanoencapsulation by miniemulsion polymerization of a renewable diester diene monomer, dianhydro-d-glucityl diundec-10-enoate (DGU), and a dithiol yielded spherical and stable nanoparticles [65]. The poly(thioether-ester) nanocapsules that were formed (particle sizes of 192–284 nm and ~98% encapsulation efficiency) contained up to 25% (*w*/*w* of DGU) of the active agent. It was observed that the monomer conversion and molar mass of the resulting polymer decreased with the increase in the amount of clove oil. The effect was tentatively ascribed to side reactions between thiyl groups and the double bond of eugenol, which could result in the quenching of the growing chains by eugenol incorporation. Moreover, a gradual reduction in the melting point from 66 °C to 50 °C was noted with the addition of clove oil. Poly(thioether-ester) nanoparticles containing 6% of clove oil exhibited an increase in antioxidant activity of ~26% for the β-carotene/linoleic acid assay and a reduction of 66% on EC_50_ for the 2,2-diphenyl-1-picryl hydrazyl assay.

Eugenol can also be encapsulated with the chitosan polymer [66]. The anticancer efficacy of the EUG-nanocapsules via the induction of autophagy was evaluated with rat C6 glioma cells. The in vitro studies involved the assessment of the drug release efficacy and its anti-metastatic properties by immunoblotting and RT-PCR analysis of epithelial–mesenchymal transition protein expression. The results proved the apoptotic potency of Eu–Cs on rat C6 glioma cells, even in the presence of an oncogenic inducer and autophagic inhibitors. The immunocytochemical studies showed the inhibition of NFκβ protein expression in the presence of Eu–Cs. The decrease in epithelial–mesenchymal transition protein expression in the treated rat C6 glioma cells proved the potential of Eu–Cs as a metastasis inhibitor.

#### 2.3.2. EUG Administration for Skin Infections and Wound Treatment

The increasing emergence of antibiotic-resistant bacterial strains like *S. aureus* and *P. aeruginosa* forces the design of new delivery systems, developed to enhance the therapeutic efficacy of existing topical anti-infectives [67]. It was shown that eugenol can facilitate the penetration of doxorubicin (Dox) into cells of *Bacillus subtilis* owing to the membrane permeability increase by the formation of defects in the bacterial wall and the inhibition of efflux proteins [68]. It makes it possible to maintain a high concentration of the antibiotic for several hours until the pathogens are completely neutralized. The regulation of efflux may be a method to overcome multiple drug resistance of both pathogenic microorganisms and cancer cells.

Nanotechnology has become a major driving force in the treatment of skin infections because it makes possible drug interaction with the skin tissue at the sub-atomic level, and vesicular nanocarriers (liposomes and nanocapsules) are currently the most promising drug delivery solutions in the treatment of topical infections. Nanoemulsions display very good biocompatibility but can also efficiently penetrate and accumulate in biofilms. Eugenol-based materials were studied for the treatment of topical infections that require the application of such efficient anti-infective molecules. Although nanoemulsions are very useful in dermal delivery systems of lipophilic drugs, topical applications require a higher viscosity of the materials containing the active agents. Hydrogel-thickened nanoemulsions (HTN) were thus prepared using chitosan as a thickening polymer in eugenol-based oil-in-water (o/w) HTN, which was applied for the transdermal delivery of 8-methoxsalen (8-MOP) [69]. Transdermal permeation of 8-MOP from EUG HTN was found to be strongly dependent on the molecular weight of chitosan.

Eugenol was also used as an antimicrobial agent and a component of biodegradable poly(oxanorbornenimide) (PONI)-based nanoemulsions, which transported silver nanoclusters (AgNCs) into biofilms [70]. Hydrophobically modified AgNCs were incorporated into the EUG-containing phase and the dual-antimicrobial Ag-BNE nanomaterials displayed broad-spectrum activity against *E. coli*, *Acinetobacter baumannii*, *P. aeruginosa*, and methicillin-resistant *S. aureus* (MRSA). A 4- to 16-fold decrease in minimum biofilm bactericidal concentrations (MBBCs) compared to the individual component species resulted from the co-delivery of eugenol and AgNCs. The synergistic action of therapeutics and the increased treatment efficacy, despite decreasing the required dosing, was attributed to the enhanced delivery of Ag ions into the biofilm. Fluorescence studies confirmed the action of the dual-mode nanoemulsions containing eugenol and AgNCs that involved penetration of the biofilm and the disruption of bacterial membranes. The nanoemulsions were not cytotoxic at concentrations effective against bacterial biofilms.

**Figure 6:**
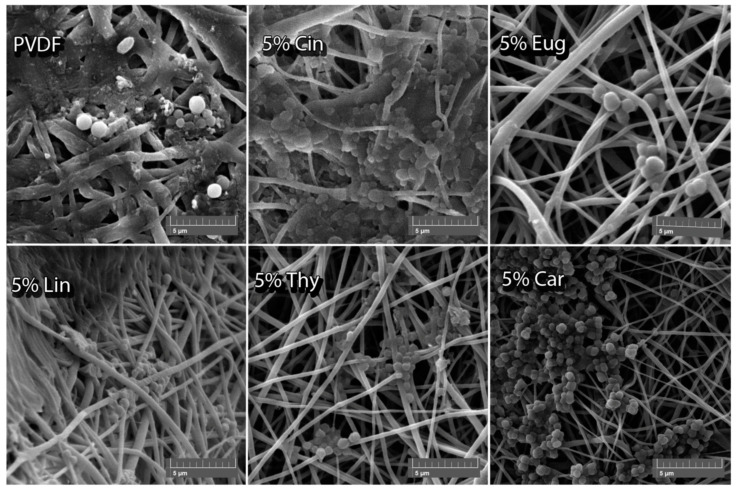
SEM images of *S. aureus* biofilm formation on pure PVDF nanofibers and samples electrospun with addition of individual EOs [71].

Polymeric electrospun nanofibers are an interesting form of nano-objects that can be used as containers for various active agents. Nanofibers have been used in healthcare and environmental technologies, including the preparation of filtration membranes in water treatment. The action of such membranes can be hampered by fouling with bacteria biofilms that clog the active surface. The enrichment of nanofibers with active molecules, including eugenol, may help to prevent these negative effects. For example, hydrophobic polyvinylidene difluoride (PVDF)-based electrospun nanofibers (diameters of 570–900 nm) with various essential oils incorporated (eugenol, thymol, linalool, cinnamaldehyde, and carvacrol) were used for membrane preparation, and their antibacterial and antibiofilm properties were tested against *Escherichia coli* ATCC 25922 and *Staphylococcus aureus* ATCC 25923 [71]. Biofilm formation on the membrane surface was effectively reduced when PVDF matrix contained thymol or eugenol (Figure 6).

Chitosan and gelatine are natural macromolecular components often used for the formation of electrospun nanofibers for wound dressing. Synthetic polymers such as polyvinyl-alcohol (PVA) can be used as the matrix or as a co-spinning agent. Thus, antimicrobial nanofibrous mats made of electrospun chitosan/cellulose acetate/gelatine (4:1:5 *v*/*v*) blended with admixed eugenol (0–10% *v*/*v*) were successfully fabricated [72]. Nanofibers with larger diameters (~150 to ~290 nm) and junctions were obtained upon increasing the concentration of EUG. Thermal stability was diminished, and temperatures of phase transitions (glass transition and melting) decreased upon increasing the amount of EUG. The burst release of eugenol from the fibers (observed within 300 min) also depended on its content. For smaller amounts of EUG (≤1.5% *v*/*v*), the burst release reached equilibrium after 1 h but gradually increased at EUG ≥ 3% *v*/*v*. The hybrid nanofibrous mats with incorporated EUG effectively hampered the growth of both *Salmonella typhimurium* and *S. aureus*. These results suggest that bio-based and biocompatible nanofibers can be applied for antibacterial textiles, active food packaging, and air filtration, as well as for wound dressing.

**Figure 7:**
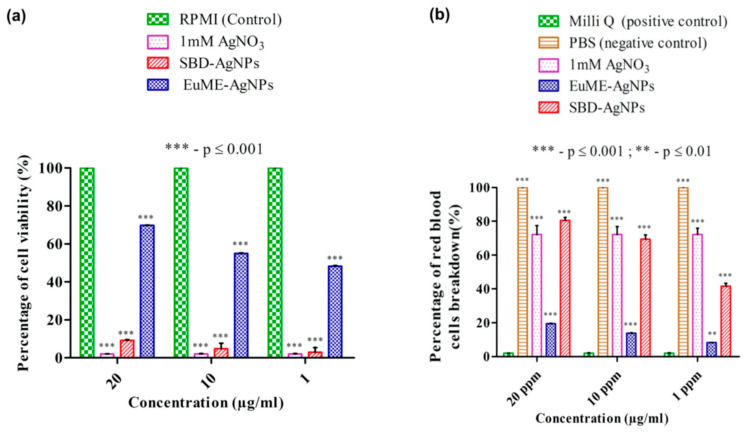
Percentage of (**a**) WBC viability with EuME–AgNPs and SBD–AgNPs and (**b**) RBC breakdown with EuME–AgNPs and SBD–AgNPs. Reproduced from Ref. [73] with permission from the Royal Society of Chemistry.

Eugenol can be also used as a component of more complex compositions containing other antimicrobial agents. Thus, EUG was employed as a microemulsion (EuME) medium for introducing silver nanoparticles (AgNPs) into polyvinyl alcohol [73]. The hybrid blend was applied to fabricate electrospun nanofibers (EuME-AgNP-NFs) that could be used for wound dressing. Homogeneous electrospun nanofibers with a uniform pore diameter of 404.1 nm and elemental silver composition of 13.93% were obtained. The antibacterial efficiency of EuME-AgNP-NFs was compared to that of silver bandaid-suspended nanoparticle nanofibers (SBD-AgNP-NFs). The results proved that the eugenol-based system can provide an environment suitable for wound healing. EuME-AgNPs exhibited effective antibacterial action against *S. aureus.* The effect was linked to the sustained release of silver ions, which was observed in the simulated wound fluid system. Studies on EuME-AgNPs’ interaction with human lymphocytes and erythrocytes revealed a 69.81% rate of cell viability and a 19.44% rate of red blood cell breakdown (Figure 7).

Biodegradable polymeric nanoemulsions that encapsulated two different hydrophobic antimicrobial agents (eugenol and triclosan) (TE-BNEs) were applied to combat chronic wound infections [74]. The action of the dual-loaded system allowed for synergistic treatment of wound biofilms. The TE-BNEs were found to be highly active (99% bacterial elimination) in an in vivo murine model of mature multidrug-resistant (MDR) wound biofilm. Moreover, application in the form of a nanoemulsion delayed the emergence of resistance to triclosan.

Synergistic action was also noted in the case of electrospun polycaprolactone nanofibers loaded with multiple natural antibacterial compounds [75]. Three-component mixtures of curcumin–piperine–eugenol (PCPiEu) and curcumin–piperine–rutin (PCPiR) were designed for this purpose. Their effect was compared with those of one- and two-component systems. The smooth and uniform nanofibers of PCPiEu were slightly larger than those of PCPiR (198.38 and 142.60 nm, respectively). Although their water absorption and water vapor permeability were suitable for wound dressing (8.33–10.42 mg cm^2^ h^−1^), the mechanical properties of the nanofibers slightly deteriorated in comparison with the control sample. Antibacterial tests showed good partial toxicity results for PCPiEu. Both PCPiEu and PCPiR exhibited antibacterial activity against G+ *S. aureus* and G- *Enterococcus faecalis* (killing ability of 74% and 75% against G+ and 99.47% and 96.88% against G- bacterium, respectively).

Wound treatment is particularly complicated in patients with diabetes. In this case, healing skin ulcers and the formation of new tissue is challenging due to the lack of macrophage and fibroblast growth factors (TGF-β1 and PDGF, respectively), which are required for the synthesis of ECM. Electrospun nanofibers have significant potential in this field as they can form bundles (via the action of hydrogen bonds and physical interactions) that resemble the morphology of ECM [76]. Co-electrospun nanofibers with unique core-shell morphology can be loaded in the core section with therapeutic compounds (including eugenol). Gradual liberation of the active species occurs via permeation or slow degradation of the outer shell.

Another method of drug administration is based on hydrogels, which have proven useful in wound healing. They can mimic the extracellular matrix by providing a moist, biocompatible environment of adaptive mechanical properties, but also allow for the incorporation of various drug molecules, including those of antibacterial action [77]. Wound dressing strategies of this type are very important since wound biofilm infections, especially those caused by multidrug-resistant (MDR) bacteria, can be highly challenging. Specific targeting of the different stages of wound healing can be achieved in a novel approach to multiphase-directed tissue regeneration. Bioactive eugenol can be used as a component of hydrogels to enhance the process.

Essential oils can also be used as substitutes for antibiotics in biomedical implant materials, where antimicrobial activity is required for good tissue regeneration. For instance, eugenol and cinnamon oil (COL) were tested against three pathogens involved in biomaterial-associated infections: *S. aureus*, *Staphylococus epidermidis*, and *E. coli*, as components of porous scaffolds with square-shaped macropores, prepared with biodegradable and biocompatible poly(ε-caprolactone) (PCL) [78]. The pure PCL and blends prepared with 30% EO (PCL/EUG and PCL/COL) were not cytotoxic. (GC-MS analysis revealed that the active concentration of EO within the scaffolds was reduced to ~10%, in the 30% EO-doped sample). Eukaryotic osteoblast-like Saos-2 cells’ viability/proliferation was hampered in contact with the CL matrix admixed with 40 and 50% of EO. PCL/30%EO scaffolds slightly reduced the growth of staphylococci (a small inhibition halo). Yet, a significant decrease was recorded on both adherent and planktonic bacteria for all three bacterial strains, indicating partial EO release to be the cause of the observed anti-adhesiveness.

#### 2.3.3. EUG in Dentistry and Treatment of Oral Diseases

**Scheme 5:**
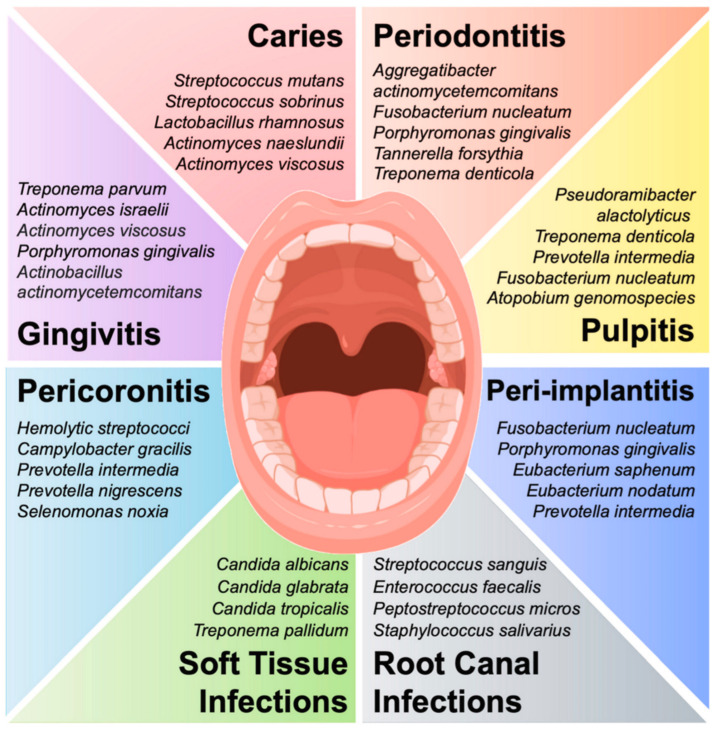
Types of oral and systemic diseases associated with pathogen microorganisms [12].

Drug delivery systems based on polymeric nanoparticles (PNPs) have been also explored for the treatment and prevention of dental biofilm-related oral diseases [79]. PNPs loaded with an active mucoadhesive drug or antimicrobial agent can provide a better effect due to the increase in drug uptake by cells in an aqueous environment. The release of drugs can be performed in a time-controlled way. A huge development of smart dental biomaterials for antimicrobial applications in dentistry can be noted. They can react to physiological stimuli (pH, enzymes) or external environmental factors (e.g., light or mechanical, electrical, and magnetic signals) [12]. Such an approach helps to prevent microbial infections and additionally may extend the longevity of dental devices. It is very important, especially as bacterial infections are the major cause of oral diseases (Scheme 5) and can adversely influence the entire organism.

4-allyl-2-methoxyphenol has been used in dentistry for several decades due to its ability to inhibit the growth of a range of microorganisms, including anaerobes commonly infecting root canals. EUG can be added as an antimicrobial agent to therapeutic oral gels and applied in situ [80].

The direct application of eugenol may result in damage to the pulp tissue. However, when eugenol is mixed with zinc oxide, an acid–base reaction occurs between phenol groups of EUG and Zn^2+^ ions, resulting in the formation of a crystalline chelate–zinc eugenolate (ZOE) [81]. Even today, the chelate is frequently used for the preparation of restorative tooth fillings, in prosthodontics, as an anesthetic, and for root canal sealing. For the latter application, the antimicrobial action of ZOE can be crucial, as the root-end filling should prevent the leakage of irritants from the root canal into the gum tissue. When ZOE is applied as an intermediate restorative material to a dental cavity, hydrolysis of zinc eugenolate occurs slowly upon exposure to aqueous media (such as oral body fluids) yielding eugenol and zinc hydroxide [81]. The liberated molecules of EUG can diffuse through dentin and into the saliva. The effects of eugenol on tissue appear to be highly dependent on its concentration. High concentrations are cytotoxic and may cause irreversible blockage of nerve conduction (a neurotoxic effect) [81]. At low concentrations eugenol exerts a beneficial anti-inflammatory effect on the dental pulp, thus facilitating its healing. Moreover, at low concentrations, eugenol can reversibly inhibit the activity of nerves, thus acting as a local anesthetic. It was also observed that EUG reduced synaptic transmission at the neuromuscular junction and inhibited prostaglandin synthesis and white cell chemotaxis.

ZOE showed relatively good biocompatibility, and its’ in vitro cytotoxicity to immortalized human oral keratinocytes (IHOKs) was attributed to the release of Zn ions (half maximal effective concentration EC_50_ of 5-44 ppm), not eugenol (EC_50_ not detectable under 100 ppm) [82]. It was proved that the anti-inflammatory response of IHOKs to ZOE (lowered levels of inflammatory IL-1β, IL-6, and IL-8 cytokine gene expressions) was induced by a combination of Zn^2+^ and eugenol. Similar results were obtained for immortalized human dental pulp stem cells (IHDPSCs) and mouse bone marrow monocytes (IMBMMs) in contact with ZOE [83]. It was shown that over the same experimental period, ZOE prevents microbial leakage much better than mineral trioxide aggregate (MTA) [84,85]. The filling effectiveness of ZOE when applied with a pressure syringe is comparable to other filling materials (Vitapex^®^, Calcicur^®^, Feapex, and Calen^®^-ZO) [86]. ZOE, Calen^®^-ZO, and Vitapex^®^ showed similar values of the total volume of the filled canal (%FC) while Feapex and Calcicur^®^ showed lower %FC.

**Figure 8:**
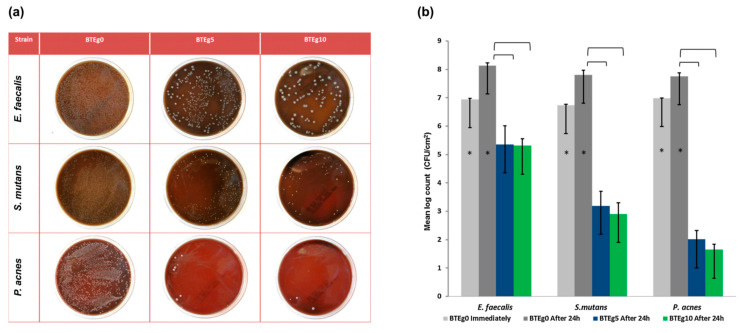
(**a**) Images of composites surface antibacterial activity assay after 24 h incubation (dilution factor −2); (**b**) the mean log_10_ CFU/cm^2^ of tested bacterial species on the experimental composites (* Significant differences observed for the control sample (BTEg0) recovered immediately and after 24 h incubation (*p* < 0.01). The horizontal lines indicate significant differences between the control sample and tests at EgMA concentration of 5 and 10% by weight, after 24 h incubation (*p* < 0.01)). Ref. [87] Copyright (2016), with permission from Elsevier.

Eugenol can be also applied in macromolecular form for the treatment of various oral diseases. A polymeric derivative of eugenyl methacrylate monomer (EgMA) was used as a component (0, 5, and 10% by weight) of dental composite formulations based on BisGMA/TEGDMA resin admixed with hydroxyapatite (HA) and zirconium oxide ZrO_2_ as fillers [87]. A reduction in water uptake was observed when increasing the content of the EgMA monomer in the composites. Correspondingly, surface free energy values decreased indicating a significant increase in their hydrophobicity. The incorporation of EgMA monomer within polymerizable formulations yielded intrinsically antibacterial resin composites that can be used for different dental applications. The in vitro behavior of the prepared materials was studied, as well as their wettability and antimicrobial action, against a range of oral bacteria (*Enterococcus faecalis*, *Streptococcus mutans*, and *Propionibacterium acnes*) (Figure 8). The composites exhibited effective bacteriostatic activity by reducing the number of CFUs of the bacteria tested with no significant dependence on the concentration of EgMA at 5 and 10% by weight. The covalent bonding of the eugenol derivative within the resin eliminated its leaching, and thus no inhibition zones were detected when the composites were tested. Nevertheless, the antibacterial activity R of their surfaces (values of 2.7–6.1) was different against the three tested species following the order *E. faecalis* < *S. mutans* < *P. acnes*.

## 3. Conclusions

This review article presents the current state-of-the-art design and advantages of eugenol-based materials for antimicrobial applications. The use of polymeric nanocarriers not only can improve the stability, bioavailability, and cellular uptake/internalization of natural compounds but also reduce their toxicity. There are a number of EUG delivery methods using polymeric materials (in the form of nanocapsules, liposomes, microemulsions, micelles, and dendrimers). Such methods have been used to prepare drug delivery systems for various types of medical treatment, including anticancer therapies. Modern systems of EUG delivery for skin and wound treatment are based on hydrogels and electrospun nanofibers. The effect of eugenol is often enhanced by the presence of active nanoparticles of metals and metal oxides (ZnO).

The prospects for the development of EUG-based macromolecular systems in the broader biomedical field are very promising, as there is a great need to develop new therapies in view of the rapidly increasing incidence of life-threatening bacterial, fungal, and viral infections. The research focused on solutions that involve incorporation of biologically active natural substances into hybrid, multicomponent polymeric nanoparticles with antimicrobial/antiviral activity systems has a great potential.

The availability of eugenol from reproducible natural sources and the general low tendency of bacteria to develop resistance to the bactericidal effects of eugenol-based polymeric materials are two strong pillars for future applications of the antimicrobial solutions presented in this review. Specific properties of eugenol and the possibility of obtaining synergistic therapeutic effects from the use of EUG combined with other bioactive agents are also of great importance. This approach may, in the future, allow the precise design of novel efficient drug delivery systems, including targeted anti-cancer therapies.

## Data Availability

Not applicable.
